# Optimized Flomoxef Dosing Strategy for Extended-Spectrum Beta-Lactamase-Producing Bacteria, Based on Pooled Pharmacokinetic and Pharmacodynamic Analysis

**DOI:** 10.3390/pharmaceutics18101297

**Published:** 2026-10-14

**Authors:** Tetsushu Onita, Yui Sano, Nami Kuruma, Kazuro Ikawa, Noriyuki Ishihara, Hiroki Tamaki, Kenta Yagi, Takahisa Yano

**Affiliations:** 1 Department of Pharmacy, Shimane University Hospital, 89-1 Enya, Izumo 693-8501, Japan; 2 Graduate School of Biomedical and Health Sciences, Hiroshima University, 1-2-3 Kasumi, Minami-ku, Hiroshima 734-8551, Japan

**Keywords:** flomoxef, ESBL, pharmacokinetics, pharmacodynamics, prolonged infusion

## Abstract

**Background**: Flomoxef exhibits antibacterial activity against extended-spectrum beta-lactamase (ESBL)-producing bacteria and is a potential alternative to carbapenems. However, its pharmacokinetic (PK) characteristics remain unclear, and the appropriate minimum inhibitory concentration (MIC) breakpoint for flomoxef remains controversial. This study aimed to develop a flomoxef PK model using pooled meta-analysis and to evaluate various dosing regimens for ESBL-producing bacterial infections. **Methods**: Flomoxef concentration data were systematically collected from PubMed and J-STAGE. PK modeling of flomoxef was performed using the collected serum concentration data with NONMEM 7.5. Patient characteristics were examined as covariates for clearance and volume of distribution. For stochastic PK/pharmacodynamic (PD) simulation, the probability of attaining the PK/PD target {70% free time above the MIC [*f*T > MIC]} was calculated. The target MIC was determined by systematically reviewing flomoxef susceptibility data for ESBL-producing bacteria across different regions. **Results**: The population PK analysis included 39 adult patients [mean age, 58.2 years; mean body weight, 52.7 kg; and mean creatinine clearance (CL_cr_), 44.1 mL/min]. In the PK model, CL_cr_ was incorporated as a covariate of flomoxef clearance. A visual predictive check and goodness-of-fit plots demonstrated relatively good stability and predictive performance of the final model. For the PD evaluation, the target MIC was set at 4 µg/mL based on the systematically collected susceptibility data for ESBL-producing bacteria. PK/PD breakpoints for dosing regimens of 1 g twice daily (2 g/day) in typical patients with CL_cr_ = 10 mL/min, 1 g three times daily (3 g/day) in patients with CL_cr_ = 30 mL/min, and 1 g four times daily (4 g/day) with 3 h-infusion in patients with CL_cr_ = 60 and 90 mL/min achieved the target MIC. **Conclusions**: Based on the stochastic PK/PD simulation results, prolonged-infusion regimens may more effectively cover isolates with a target MIC of 4 µg/mL than short-duration infusion regimens in typical patients with normal renal function (CL_cr_ = 60 or 90 mL/min). However, clinical studies in various scenarios including severe infections are needed to identify the relationship between PK/PD results and clinical efficacy since our findings are based solely on simulation.

## 1. Introduction

Extended-spectrum beta-lactamase (ESBL)-producing bacteria (e.g., *Escherichia coli* and *Klebsiella pneumoniae*) are clinically significant pathogens that are resistant to various antibiotics. Carbapenems have been used as first-line therapy for infections caused by ESBL-producing bacteria because their clinical efficacy against these pathogens is superior to that of other antibiotics. However, carbapenem resistance has become a major concern in recent years [1], creating a need for alternative treatment options to limit the further emergence of carbapenem-resistant bacteria. Flomoxef, an oxacephem antibiotic, exhibits antibacterial activity against ESBL-producing bacteria and, together with cefmetazole, may serve as an alternative to carbapenems [2].

From a pharmacokinetic/pharmacodynamic (PK/PD) perspective, the antibacterial activity of cephems such as flomoxef largely depends on the duration for which blood concentrations remain above the minimum inhibitory concentration (MIC) of the pathogen (T > MIC) [3]. Several studies have characterized the population PK of flomoxef in adult patients [4,5]. However, because flomoxef is used primarily in Asian countries such as Japan, China, Malaysia, and Taiwan, available PK data remain limited.

Furthermore, the susceptibility of ESBL-producing bacteria varies among regions [2,6,7,8,9,10,11,12,13,14,15,16], and PK/PD evaluations integrating susceptibility data from multiple countries have not yet been conducted.

This study aimed to develop a flomoxef PK model using pooled meta-analysis and to propose dosing strategies for ESBL-producing bacterial infections by integrating regional differences in antimicrobial susceptibility.

## 2. Materials and Methods

### 2.1. PK Data Extraction

Clinical PK studies were systematically identified using the PubMed and J-STAGE databases with the search terms “flomoxef” AND “clinical” NOT “neonate.” (Search date: 1 March 2025) Studies reporting individual flomoxef concentration data in adult patients were screened. Studies for which individual PK data or patient background information were unavailable were excluded. Individual row data for total blood concentrations (written numbers, not digitized values) and administration details (dosage, infusion duration) reported in the literature were reflected in the dataset. Missing data on patient background (age and body weight) were imputed using the median. For creatinine clearance (CL_cr_) values below 5 mL/min, a value of 2.5 mL/min was assigned.

### 2.2. Population PK Modeling

A population PK analysis was performed using the collected serum flomoxef concentrations with NONMEM (version 7.5; ICON Public Limited Company, Dublin, Ireland). First, the base structural model was evaluated using one- and two-compartment models. Inter-individual variability was set using an exponential error model as follows. First, we used θ_i_ = θ × exp(η_i_), where θ_i_ is the fixed-effects parameter for the i-th subject, θ is the mean value of the fixed-effects parameter in the population, and η is a random inter-individual variable, which is normally distributed with mean zero and variance ω^2^. As residual variability, base models were examined for the additive, proportional, and combined (additive and proportional error models were investigated to describe intra-individual variation).

Second, to identify whether covariates should be incorporated into the model, specific patient characteristics (e.g., age, body weight, and CL_cr_) were tested for whether they correlated with individual-predicted PK parameters estimated from a base model. The correlation of age, body weight or CL_cr_ with clearance were examined. The correlation of body weight with volume of distribution was examined. Regarding the covariate analysis for clearance, given the intercorrelations among age, body weight, and CL_cr,_ we evaluated the incorporation of the covariate showing the strongest correlation. CL_cr_ was then calculated using the Cockcroft and Gault equation. The covariates model was expressed as follows:

CL_i(k)_ = CL_(k)_ × (x_i_/median(x))^θx^

Here, CL_i(k)_ represents the model-predicted parameter for analyte k for a typical individual i with covariate xi. CL_(k)_ represents the population central tendency for individual CL_i(k)_. Finally, the median(x) represents the median of the covariate of the subjects and θ_x_ represents a scale factor.

### 2.3. Model Qualification

To evaluate the robustness of the final model, a non-parametric bootstrap analysis was performed using Perl-speaks-NONMEM software (version 5.4.0) [17]. The final parameter estimates were compared with the 95% confidence intervals derived from 1000 bootstrap replicates. The predictive performance of the final model was assessed using goodness-of-fit plots. In addition, a visual predictive check was performed to evaluate model predictability. One thousand datasets were simulated using the final parameter estimates.

### 2.4. Stochastic PK/PD Simulation

Fixed-effects parameters θ_i_ were randomly generated 1000 times using the $SIMULATION command in NONMEM according to the mean value of each parameter and the inter-individual variance. For each dosing regimen, the time at which free flomoxef concentrations reached a specific MIC (0.125−64 μg/mL) was determined, and the free time above the MIC (*f*T > MIC) was calculated as a cumulative percentage over 24 h. Free concentrations were simulated using the fixed-effects parameters and a non-protein-bound fraction (free fraction [*f*]) of 65% [18]. Regarding the protein binding rate of flomoxef, previous reports indicate a value of 35.5 ± 0.5% [19], and this value range is small (variation, 0.0141; 95% confidence, 34.5−36.5%). Therefore, a fixed protein binding of 35%, that is, a fixed unbound fraction of 65%, in our evaluation was considered to be appropriate. At each MIC, the probability of target attainment (%) was defined as the proportion of 1000 estimates achieving 70% *f*T > MIC, the bactericidal target [20]. Considering that the concentration of unbound flomoxef determines its antimicrobial activity and considering the PK/PD parameters from previous studies on flomoxef dosing regimens for ESBL-producing bacteria [21], we adopted 70% *f*T > MIC as the target index. Although Tashiro et al. defined the bactericidal target for ESBL-producing *E. coli* as 40% *f*T > MIC [22], their study was limited to *E. coli*. Since their lower target might underestimate the results for other species such as *Klebsiella* species and *Enterobacter* species, we conducted simulations based on the target of 70% *f*T > MIC.

### 2.5. Microbiological Data Collection for ESBL-Producing Bacteria

Microbiological susceptibility data for flomoxef against ESBL-producing bacteria were systematically identified in PubMed using the terms “flomoxef” and “ESBL.” Studies were included if they (1) reported the number of clinical isolates, bacterial species, and the flomoxef minimum inhibitory concentrations for 50% or 90% of clinical isolates (MIC_50_ or MIC_90_); (2) identified the country in which the isolates were collected; (3) involved human clinical isolates; and (4) were published between 2015 and 2025. ESBL genotype information was also collected when available. The PK/PD target MIC for ESBL-producing bacteria was determined by analyzing the collected susceptibility data.

## 3. Results

### 3.1. PK Data Extraction

Six studies reporting flomoxef serum concentration data in adult patients were identified [23,24,25,26,27,28] (Table S1). The process from literature identification to subject selection is shown in Figure S1. Four patients were excluded due to unknown administration methods. A total of 219 serum concentration measurements from 39 patients were used to develop the population PK model. Patient characteristics are presented in Table 1.

### 3.2. Population PK Modeling

**Figure 1:**
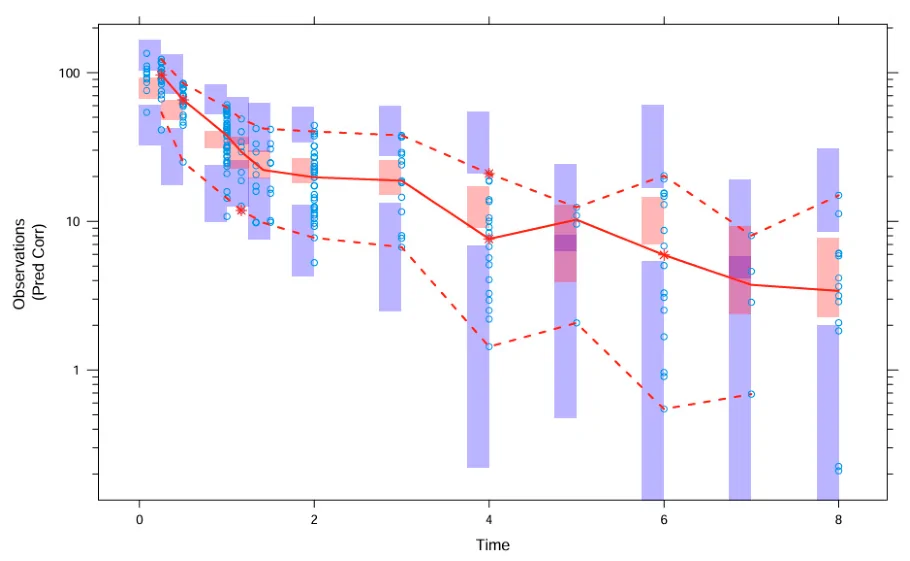
Visual predictive check plot. A visual predictive check plot representing prediction-corrected (Pred Corr) concentration for flomoxef in serum vs. time (h). Each panel shows observed 10, 50 and 90th percentile points (red lines), and predicted 90% confidence intervals for 10, 50 and 90th percentile points (blue and red areas).

**Figure 2:**
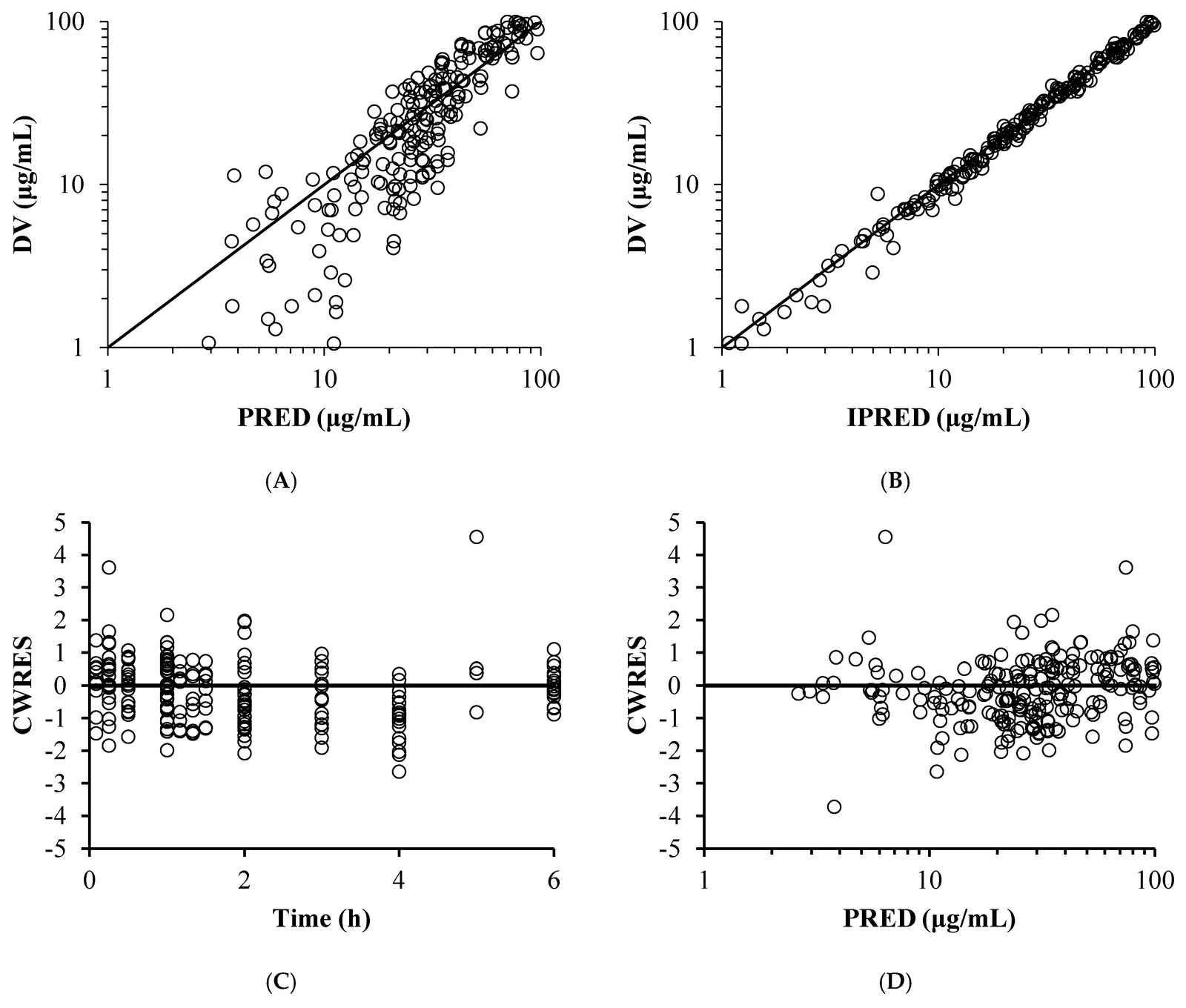
Diagnostic scatter plots of population pharmacokinetic model for flomoxef in adults. Scatter plots of the observed serum concentrations (DV) vs. population predicted concentrations (PRED) (**A**) and individual predicted concentrations (IPRED) (**B**), with the concordance lines (Y = X). Scatter plots of conditional weighted residuals (CWRES) vs. time (**C**) and PRED (**D**).

The 219 observations of total flomoxef concentration (0.14–162 µg/mL) versus time (0.083–8 h) data were best described by a two-compartment model. The final model parameters are presented in Table 2. CL_cr_ (range: 2.5–193.4 mL/min) was incorporated as a covariate of CL because it showed the strongest correlation (Figure S2), while body weight was not as a covariate of V_central_ (Figure S2 and Table S2). All parameter estimates were within the 95% confidence intervals obtained by bootstrap analysis. A visual predictive check comparing observed and predicted concentrations over time was performed (Figure 1). Visual predictive checks by renal function and data-source literature are shown in Figure S3. The observed concentrations at the 10th, 50th, and 90th percentiles were within the corresponding predicted 90% confidence intervals. Overall, the final model adequately predicted flomoxef serum concentrations. Goodness-of-fit plots are presented in Figure 2. Plots of observed concentration vs. population-predicted concentration (PRED) and individual-predicted concentration showed no major bias. Plots of conditional weighted residuals vs. PRED and time revealed no systematic trends.

### 3.3. PK/PD Target MIC for ESBL-Producing Bacteria

Twelve studies reporting flomoxef susceptibility data for ESBL-producing bacteria were identified [2,6,7,8,9,10,11,12,13,14,15,16] (Table S3). The study selection process is shown in Figure S4. Across the selected studies, MIC_50_ values ranged from 0.06 to 4 µg/mL, whereas MIC_90_ values ranged from 0.25 to 256 µg/mL. Among studies reporting ESBL genotype information, CTX-M types predominated.

### 3.4. Stochastic PK/PD Evaluation

**Figure 3:**
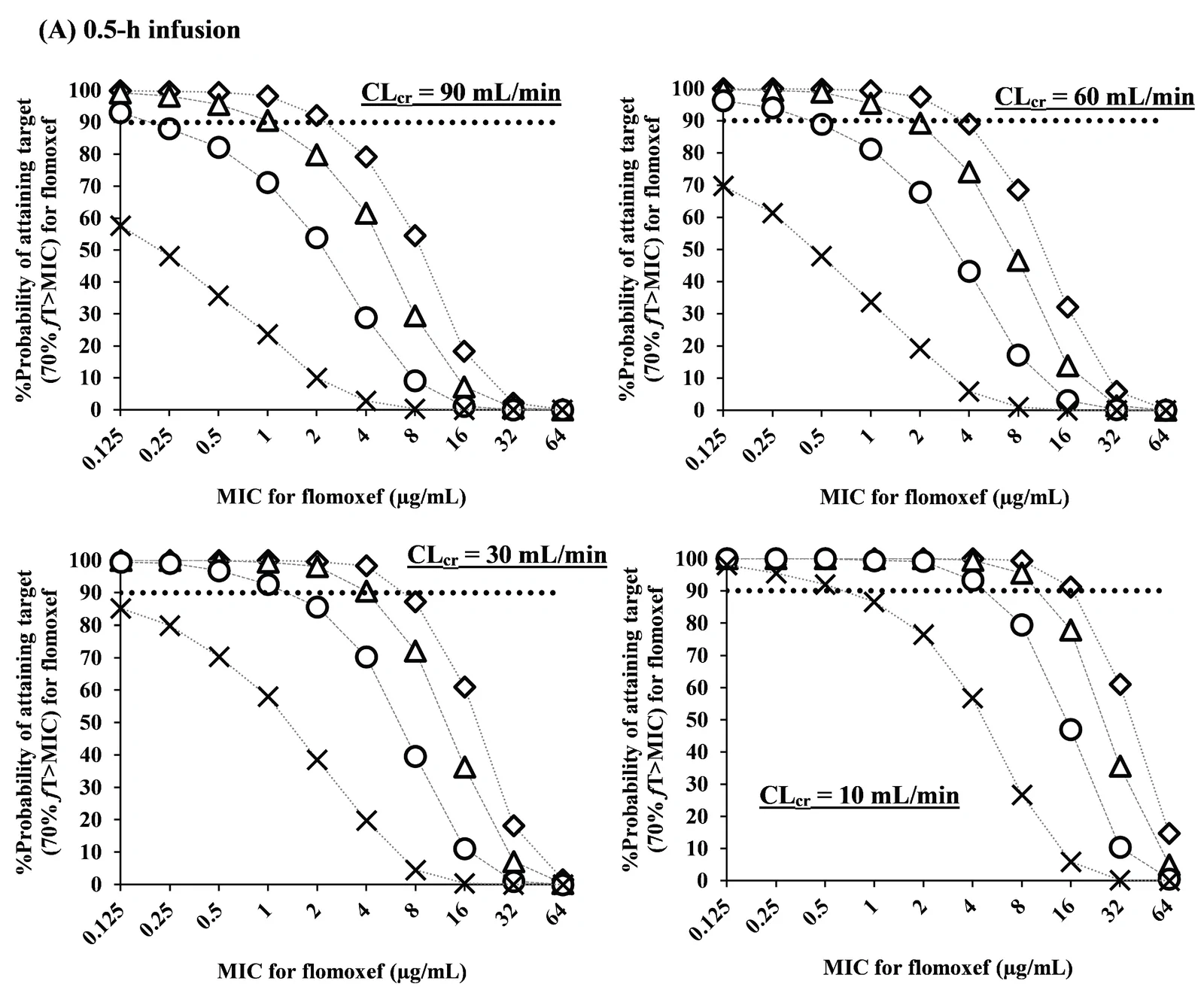

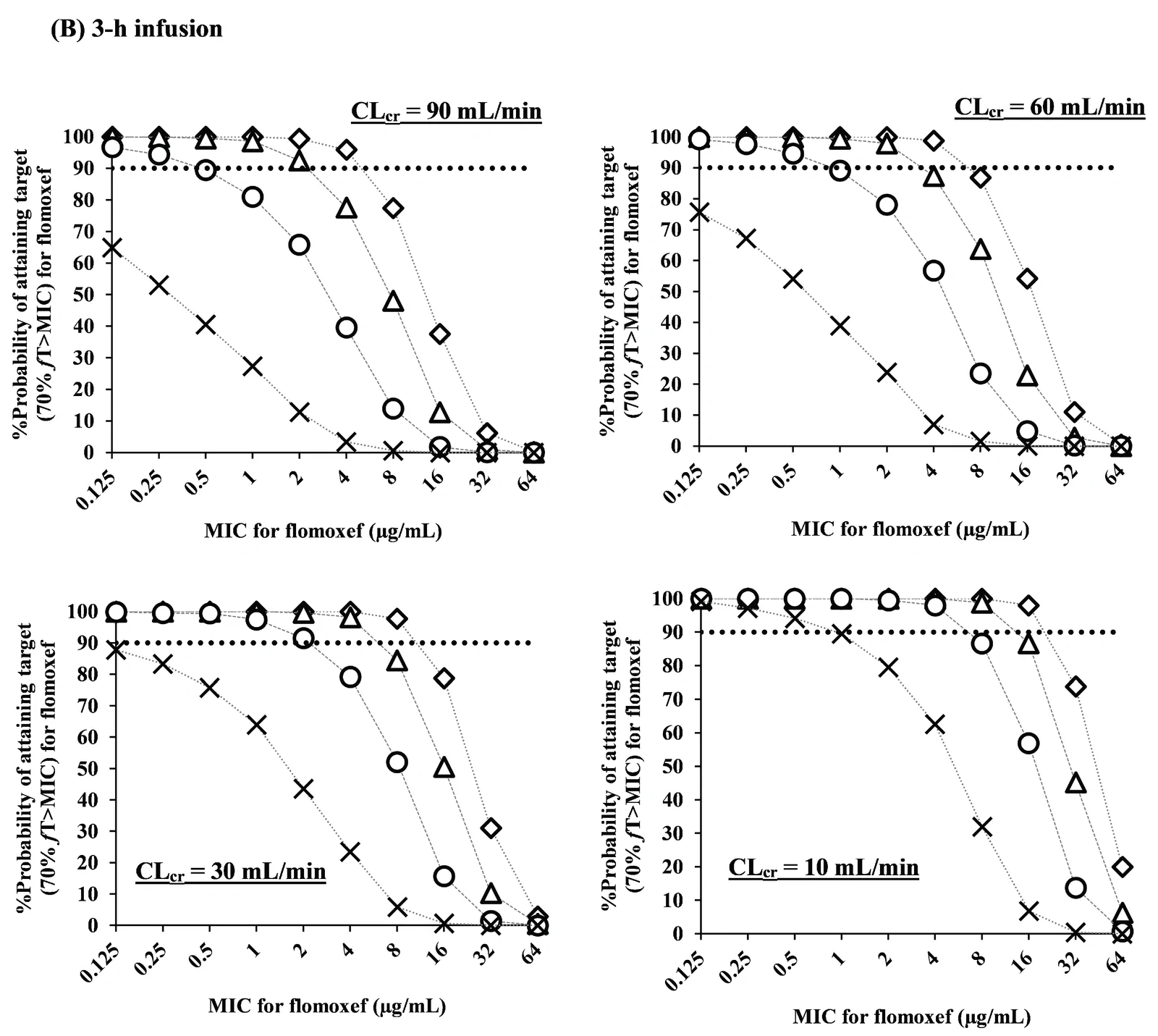
Probabilities of attaining the bactericidal target (70% *f*T > MIC) for typical patients with different creatinine clearance (10, 30, 60 and 90 mL/min), with 0.5 h (**A**) and 3 h infusion (**B**) dosing regimens (× 1 g once daily (1 g/day); ○ 1 g twice daily (2 g/day); △ 1 g three times daily (3 g/day); ◇ 1 g four times daily (4 g/day)).

The probabilities of attaining the bactericidal target at specific MICs with different flomoxef regimens (1 g once daily, twice daily, three times daily, or four times daily, administered as 0.5 h or 3 h infusions) are presented in Figure 3. The PK/PD breakpoints, defined as the highest MICs at which the target-attainment probability was ≥90%, are presented in Table 3.

With 0.5 h infusion regimens, a PK/PD breakpoint of 4 µg/mL was achieved with 1 g twice daily in patients with CL_cr_ = 10 mL/min and with 1 g three or four times daily in patients with CL_cr_ = 30 mL/min.

With 3 h infusion regimens, a PK/PD breakpoint of 4 µg/mL was achieved with 1 g twice daily in patients with CL_cr_ = 10 mL/min; 1 g three or four times daily in patients with CL_cr_ = 30 mL/min; and 1 g four times daily in patients with CL_cr_ = 60 or 90 mL/min.

## 4. Discussion

Previous reports on population PK models of flomoxef have been limited, and its PK characteristics remain incompletely defined. In addition, appropriate PD indices require further evaluation because the antimicrobial susceptibility of ESBL-producing bacteria varies among countries and regions. In this study, we developed a population PK model of flomoxef and evaluated various dosing regimens for ESBL-producing bacterial infections while considering systematically collected susceptibility data.

Regarding the population PK analysis, all flomoxef concentration–time data were best described by a two-compartment model. Based on the covariate analysis, CL_cr_ was incorporated as a covariate of CL (Figure S2 and Table S2). Because flomoxef is primarily eliminated through the kidneys, incorporation of CL_cr_ as an index of renal function into CL was considered appropriate. The estimated mean CL in our model was 4.39 L/h in a population with a mean CL_cr_ of 44 mL/min. Nakamura K et al. reported a mean flomoxef CL of 9.69 L/h in populations with mean CL_cr_ values of 66 mL/min (500 mg dose) and 61 mL/min (1000 mg dose) [4]. Komatsu T et al. reported a mean flomoxef CL of 6.25 L/h in a population with a mean CL_cr_ of 67 mL/min [5]. The CL estimated by our model was lower than those reported in these two previous models. This difference may reflect the lower renal function of our model population (mean CL_cr_ = 44 mL/min) relative to the populations in the two previous studies (approximately CL_cr_ = 60 mL/min). Therefore, our model may allow PK prediction in patients with severe renal dysfunction. For model validation, a visual predictive check and goodness-of-fit plots demonstrated relatively good stability and predictive performance of the model (Figure 1 and Figure 2). These findings indicate that the model had adequate predictive performance for subsequent PK/PD simulations. However, from the results of visual predictive checks by renal function (Figure S3A), model-predicted values for typical patients with a creatinine clearance of 90–120 mL/min tended to be lower than observed values. Therefore, the PK/PD simulations in this study were conducted for creatinine clearance values up to 90 mL/min. Based on the final model, stochastic PK/PD evaluation was performed for various dosing regimens used to treat ESBL-producing bacterial infections. The PK/PD results proposed 4 µg/mL as the target MIC for ESBL-producing bacteria. Flomoxef is considered susceptible at ≤8 µg/mL according to the CLSI breakpoints [2,6,8,10,11,12,13,16]. However, this 8 µg/mL is determined from moxalactam (another oxacephem), and is a higher breakpoint than the actual one based on the systematically collected susceptibility data (Table S3). Our 4 µg/mL encompassed the reported MIC_90_ values in 7 of 10 datasets (Table S3A), 4 of 7 (Table S3B), 2 of 2 (Table S3E), and 70.1% of ESBL-producing *E. coli*, *K. pneumoniae* and *Proteus* species isolates (MIC distribution data [9]; Table S3D). Thus, we consider 4 µg/mL to be clinically relevant value as the target MIC. Considering PK/PD breakpoints by renal function (Table 3), dosing regimens of 1 g twice daily (2 g/day) in typical patients with CL_cr_ = 10 mL/min, 1 g three times daily (3 g/day) in patients with CL_cr_ = 30 mL/min, and 1 g four times daily (4 g/day) with 3 h-infusion in patients with CL_cr_ = 60 and 90 mL/min might be reasonable from the perspective of renal dose adjustments, accumulation and safety issues. These findings suggest that prolonged-infusion regimens should be considered particularly for patients with preserved or high renal function. Consistent with this approach, international consensus recommendations support prolonged infusion of beta-lactam antibiotics, and the clinical utility of prolonged beta-lactam infusion has been increasingly recognized worldwide [29].

Because no MIC index of susceptibility for flomoxef has been established by CLSI or EUCAST, some studies have adopted the CLSI breakpoint of ≤8 µg/mL (from moxalactam but not flomoxef) for susceptibility [2,6,8,10,11,12,13,16]. However, according to our PK/PD simulation results, achieving this breakpoint (8 µg/mL) is difficult even with prolonged infusion. Therefore, flomoxef dosing should be optimized according to the MIC of the target ESBL-producing bacteria.

A previous study comparing treatment outcomes in patients with ESBL-producing bacterial infections receiving flomoxef or carbapenems as definitive therapy found that definitive flomoxef therapy appeared inferior to carbapenems for bacteremia caused by ESBL-producing bacteria, particularly for isolates with flomoxef MICs of 2–8 µg/mL [30]. In that study, flomoxef was administered at 1 g three times daily, with dose adjustment for renal insufficiency. In our stochastic simulation, the PK/PD breakpoints for 1 g three times daily administered by 0.5 h infusion were 0.5–1 µg/mL in patients with normal renal function (CL_cr_ = 60 or 90 mL/min). Therefore, optimized prolonged-infusion regimens may be required for isolates with flomoxef MICs of 2–8 µg/mL.

This study has several limitations. First, although flomoxef concentration data were collected as systematically as possible, the study population had relatively impaired renal function (mean CL_cr_ = 44 mL/min). Therefore, the evaluated dosing regimens may result in underdosing in patients with augmented renal clearance. Second, since our findings are based solely on simulation, clinical studies are needed to determine the relationship between PK/PD simulation results and clinical efficacy.

## 5. Conclusions

Based on the stochastic PK/PD simulation results, prolonged-infusion regimens may more effectively cover isolates with MIC of 4 µg/mL than short-duration infusion regimens in typical patients with normal renal function (CL_cr_ = 60 or 90 mL/min). However, clinical studies in various scenarios including severe infections are needed to identify the relationship between PK/PD results and clinical efficacy, since our findings are based solely on simulation.

## Figures and Tables

**Table 1:** Patient backgrounds.

	N = 39
	Number (%) or Mean ± S.D. (Range)
Sex (male:female)	24:15
Age (years)	58.2 ± 16.9 (19–85) ^a^
Body weight (kg)	52.7 ± 12.6 (26–92) ^b^
Creatinine clearance (mL/min)	44.1 ± 36.5 (2.5–193.4)
Underlying diseases	
Lung cancer	9 (23.1%)
Pyelonephritis	3 (7.7%)
Pneumonia	1 (2.6%)
Others	3 (7.7%)
None (healthy)	4 (10.2%)
Unknown	19 (48.7%)
Dosage (mg)	
0.5 g	9 (23.1%)
1.0 g	29 (74.4%)
2.0 g	1 (2.5%)
Administration	
Bolus	29 (74.4%)
1 h infusion	10 (25.6%)

^a^ One data point was lacking; ^b^ Six data points were lacking.

**Table 2:** Population pharmacokinetic parameters for flomoxef in adults.

Parameter	Flomoxef		
	Estimate	(RSE%)	95%CI
Fix effects parameter			
CL (L/h) = *θ*_CL_ × (CL_cr_/36.9 ^a^)^θCLcr on CL^			
*θ*_CL_(L/h)	4.39	(12.7)	3.15–6.04
*θ*_CLcr on CL_	0.474	(35.4)	0.0329–0.696
V_central_ (L) = *θ*_Vcentral_	8.93	(15.1)	6.70–11.06
Q (L/h) = *θ*_Q_	9.68	(13.1)	7.49–12.29
V_peripheral_ (L) = *θ*_Vperipheral_	8.88	(15.1)	5.76–10.98
Inter-individual variability (exponential error model)			
η_CL_	0.208	(45.3)	0.0820–0.688
η_Vcentral_	0.160	(68.8)	0.00141–0.315
η_Q_	0.0919	(67.9)	0.00989–0.341
η_Vperipheral_	0.797	(30.2)	0.230–1.26
Residual variability (proportional error model)			
ε	0.0120	(36.5)	0.00407–0.0196

CI, confidence interval determined from 1000 bootstrap replicates (success rate: 97%); RSE, relative standard error; θ, population mean value; η, random variable which is normally distributed with a mean of zero and variance; ε, random error which is normally distributed with a mean of zero and variance. a. Median as creatinine clearance in 39 subjects.

**Table 3:** Pharmacokinetic/pharmacodynamic breakpoints for bactericidal target (70% *f*T > MIC), using different dosing regimens.

Flomoxef Dosing Regimens	Pharmacokinetic/Pharmacodynamic Breakpoint (μg/mL)			
	CL_cr_ = 90 mL/min	CL_cr_ = 60 mL/min	CL_cr_ = 30 mL/min	CL_cr_ = 10 mL/min
0.5 h infusion				
1 g once daily (1 g/day)	-	-	-	0.5
1 g twice daily (2 g/day)	0.125	0.25	1	4
1 g three times daily (3 g/day)	1	1	4	8
1 g four times daily (4 g/day)	2	2	4	16
3 h infusion				
1 g once daily (1 g/day)	-	-	-	0.5
1 g twice daily (2 g/day)	0.25	0.5	2	4
1 g three times daily (3 g/day)	2	2	4	8
1 g four times daily (4 g/day)	4	4	8	16

Pharmacokinetic/pharmacodynamic breakpoints are defined as the largest MICs attained at which the target-attainment probabilities are ≥90%. Areas where the PK/PD breakpoint achieved an MIC of 4 µg/mL are shaded in gray.

## Data Availability

Data supporting the findings of this study were derived from the resources available in the public domain. The PK dataset used in this study is available from the corresponding author upon reasonable request.

## Supplementary Materials

The following supporting information can be downloaded at https://www.mdpi.com/article/10.3390/pharmaceutics18101297/s1, Figure S1: Extraction of flomoxef pharmacokinetic (PK) articles and selection of subjects for pooled PK analysis; Figure S2: Regression analysis between individual-predicted PK parameters and covariates; Figure S3 Visual predictive plots by renal function (A) and data-source literature (B), Figure S4: Microbiological data collection for ESBL-producing bacteria; Table S1: Details of selected literature and number of subjects for developing population PK model; Table S2: The results of likelihood ratio test in covariate analysis; Table S3: Details of selected articles for the susceptibility of ESBL-producing bacteria.

## Abbreviations

The following abbreviations are used in this manuscript:

CL_cr_	Creatinine clearance
CL	Flomoxef clearance
CWRES	Conditional weighted residuals
DV	Observed serum concentrations
ESBL	Extended-spectrum beta-lactamase
IPRED	Individual predicted concentrations
MIC	Minimum inhibitory concentration
PRED	Population-predicted concentration
PK	Pharmacokinetic
PD	Pharmacodynamic
Q	Central–peripheral intercompartmental clearance
V_central_	Volumes of distribution volumes of the central
V_peripheral_	Volumes of distribution volumes of the peripheral
