# Supplementary material for: Optimized Flomoxef Dosing Strategy for Extended-Spectrum Beta-Lactamase-Producing Bacteria, Based on Pooled Pharmacokinetic and Pharmacodynamic Analysis
Source: Pharmaceutics. 2026 Oct 14;18(10):1297. doi: 10.3390/pharmaceutics18101297 (PMC13708099; doi:10.3390/pharmaceutics18101297)
Supplement: Supplementary file 1 [file pharmaceutics-18-01297-s001.zip › pharmaceutics-4588205-supplementary.pdf]

Figure S1

Extraction of flomoxef pharmacokinetic (PK) articles and selection of subjects for pooled PK analysis

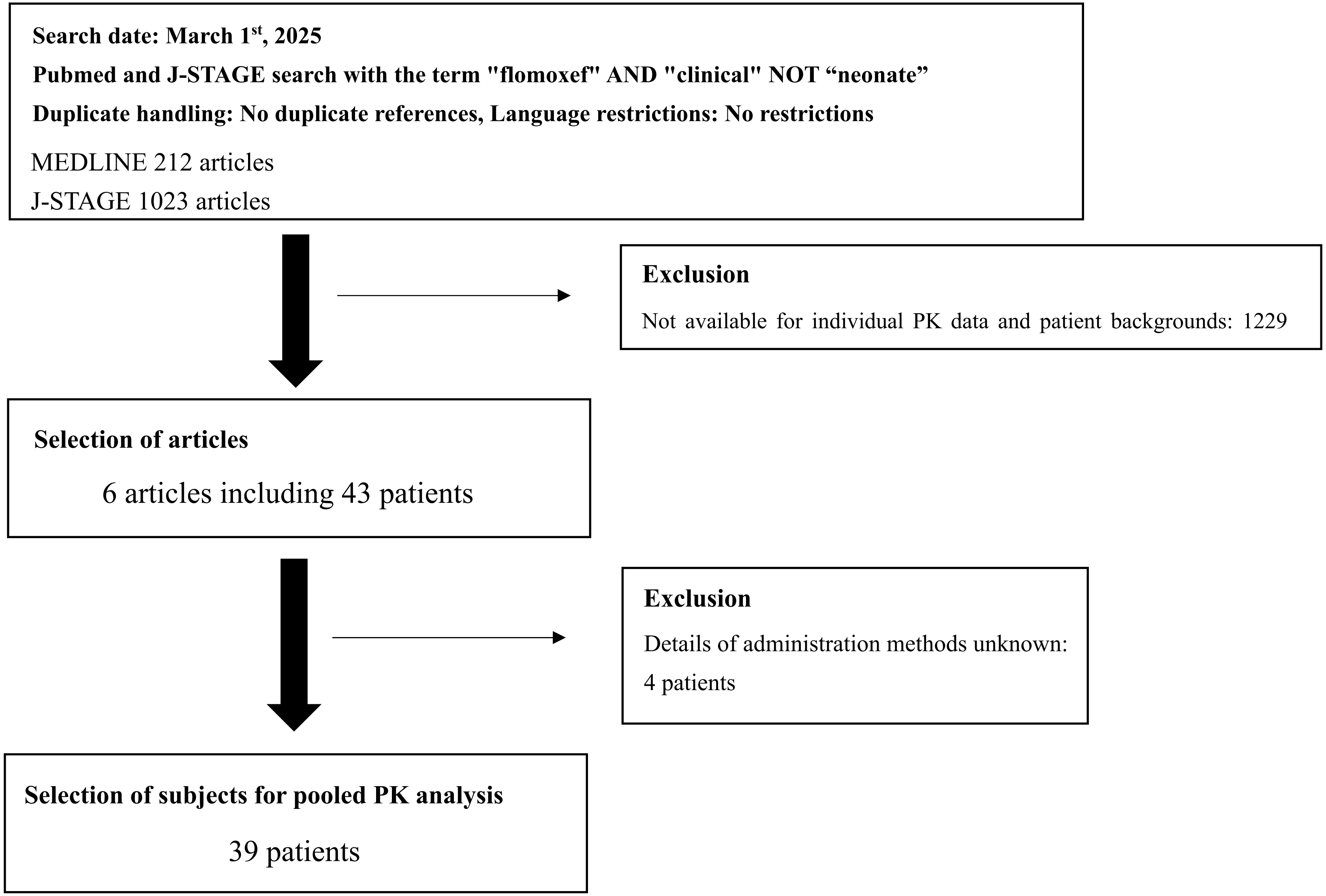

Figure S2

Regression analysis between individual-predicted PK parameters and covariates

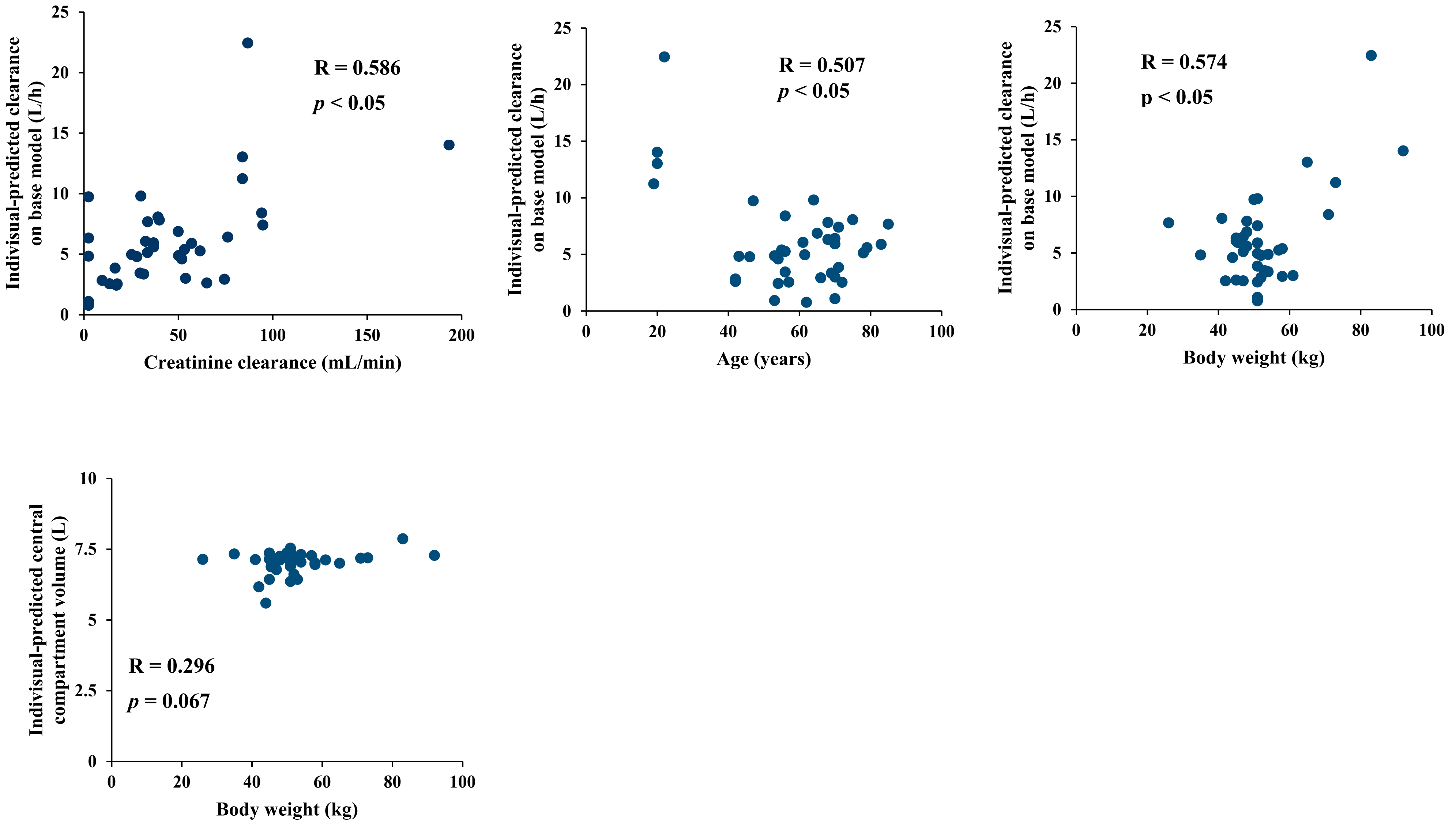

Figure S3 Visual predictive plots by renal function (A) and data-source literature (B)

(A) Visual predictive plots by renal function

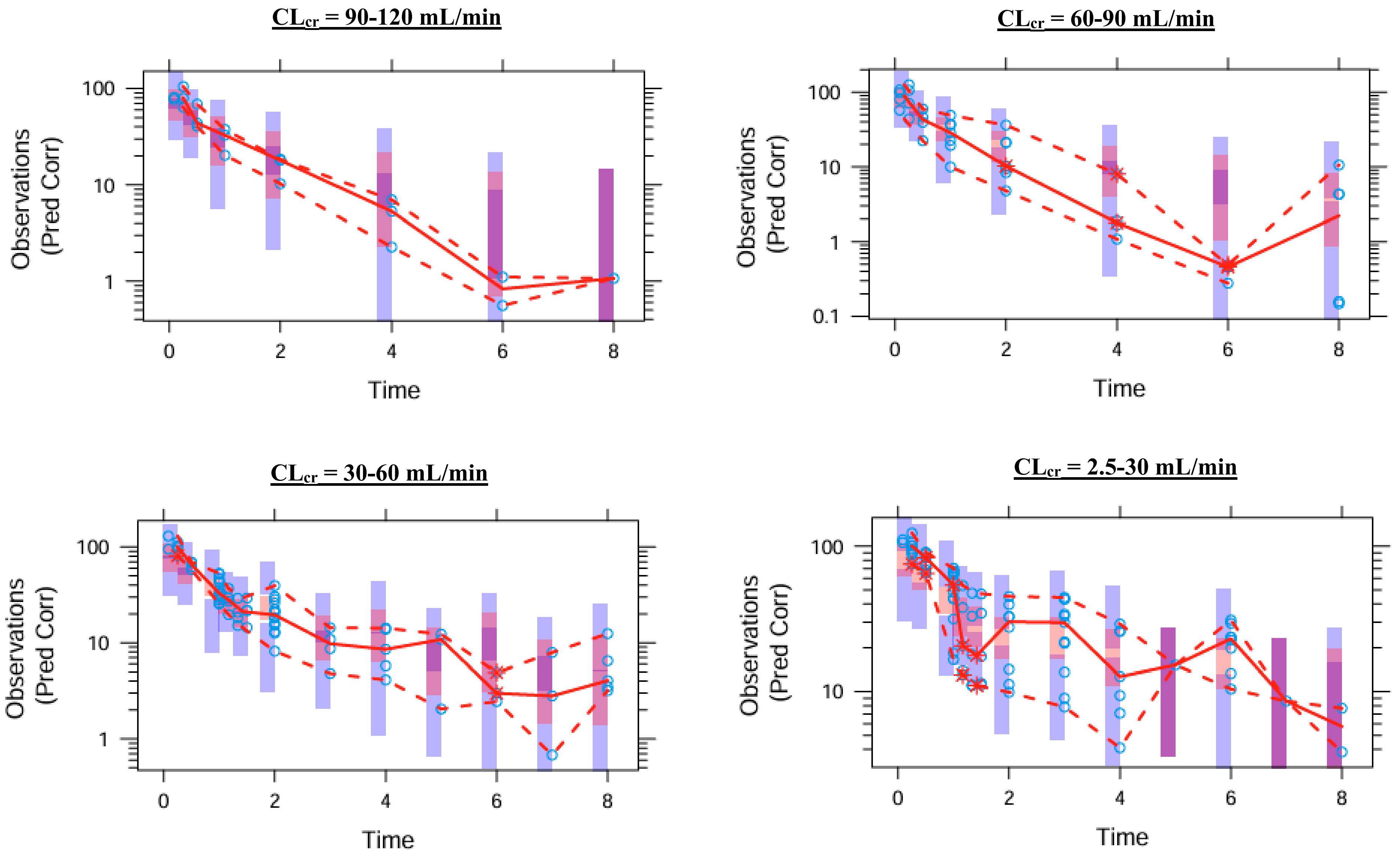

(B) Visual predictive plots by data-source literature

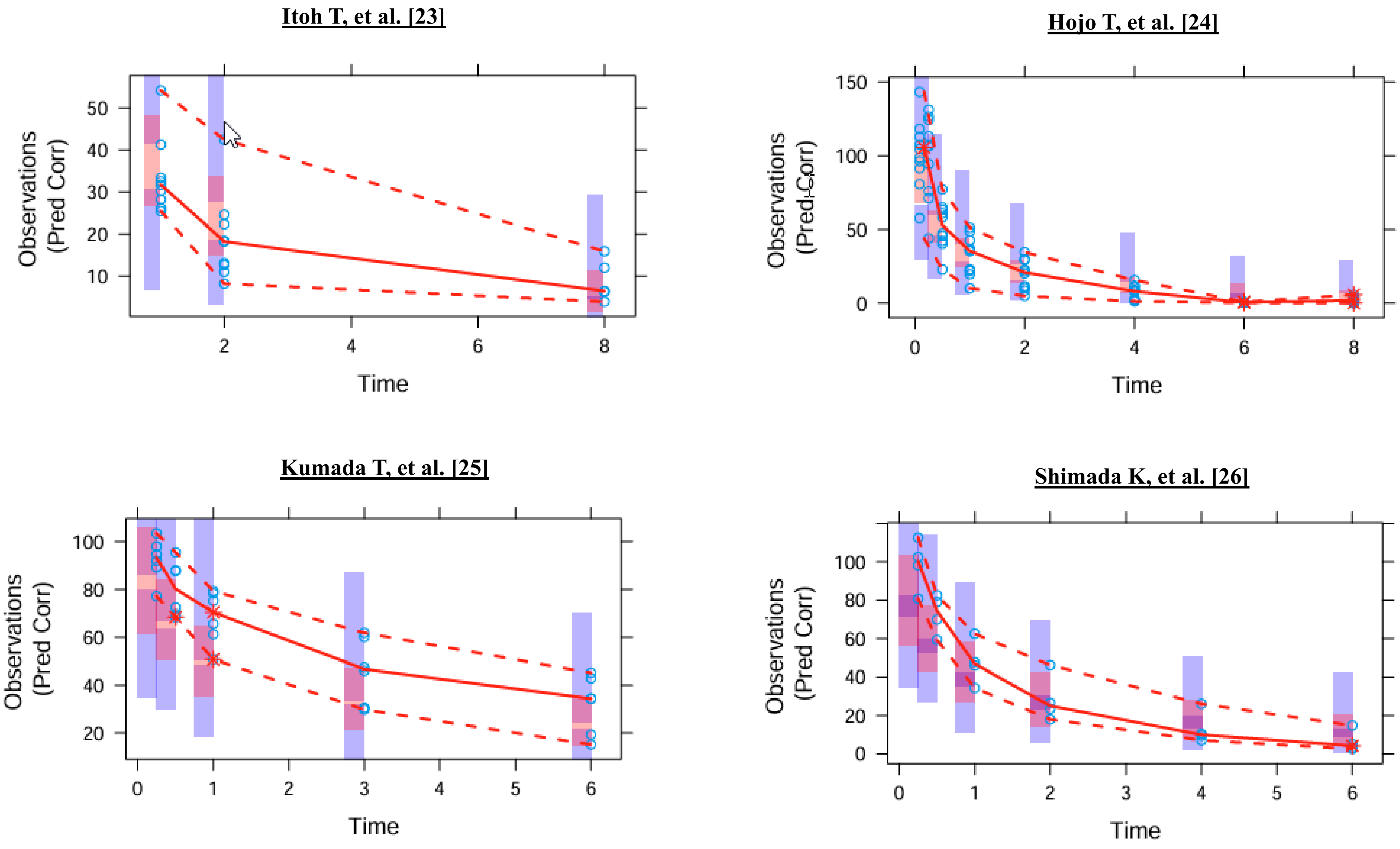

Kanimoto Y, et al. [28]

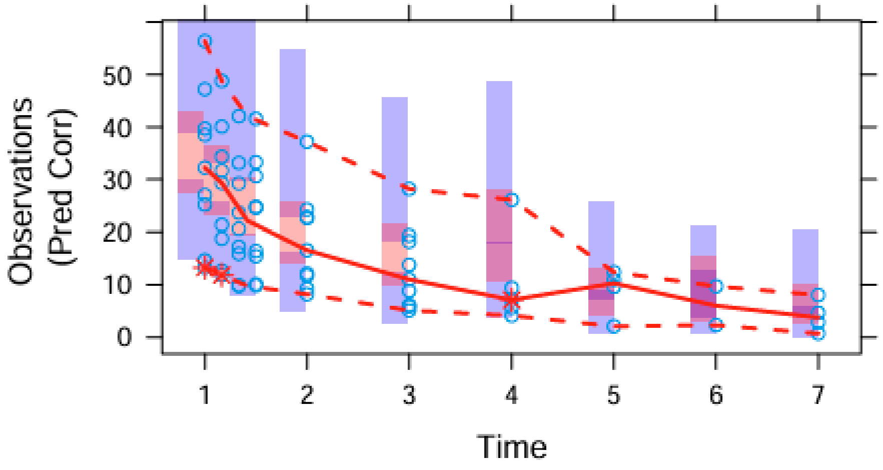

Figure S4
Microbiological data collection for ESBL producing bacteria

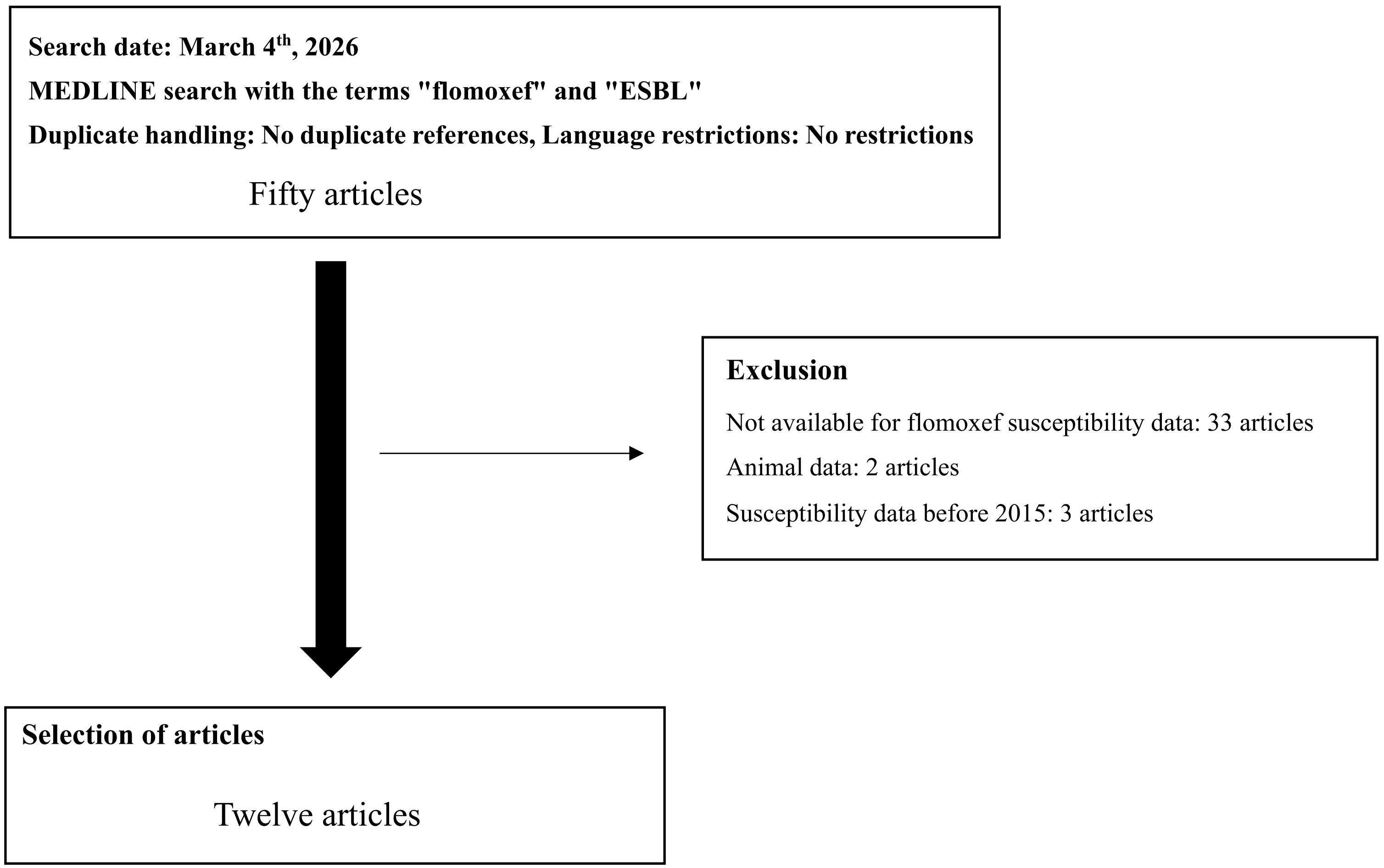

Table S1
Details of selected literature and number of subjects for developing population PK model

Literature	N
Itoh T. *Jpn J Chemother.* 1987; 35(S-1): 599-603. [23]	9
Hojo T, et al. *Jpn J Chemother.* 1987; 35(S-1): 613-626. [24]	10
Kumada T, et al. *Jpn J Chemother.* 1987; 35(S-1): 632-637. [25]	6
Shimada K, et al. *Jpn J Chemother.* 1987; 35(S-1): 649-655. [26]	4
Nagatake T, et al. *Jpn J Chemother.* 1987; 35(S-1): 727-738. [27]	1[a]
Kanimoto Y, et al. *Jpn J Chemother.* 1987; 35(S-1): 1050-1058. [28]	9

a. Four out of 5 subjects were excluded because details of the administration methods were unknown.

Table S2
The results of likelihood ratio test in covariate analysis

Models	OFV	△OFV	*p* value
**Base model**	**1011.4**	**-**	**-**
**CL - CL_{cr}**	**1009.8**	**1.57**	**< 0.05**
**$V_{central}$ - BW**	**1032.3**	**-**	**-**

OFV, objective function value; CL, clearance; $V_{central}$, Volumes of distribution volumes of the central

Table S3
Details of selected articles for the susceptibility of ESBL producing bacteria

(A) Susceptibility of ESBL producing *Escherichia coli*

Literature	N	MIC_{50}	MIC_{90}	MIC range	ESBL genotype
Yang Q, et al. 2015, China [2]	59	0.125	1	NA	$bla_{CTX-M-14}$ (50.8%), $bla_{CTX-M-15}$ (35.6%), $bla_{CTX-M-55}$ (23.7%), $bla_{CTX-M-65}$ (3.4%)
Yang Q, et al. 2015, China [6]	196	0.125	0.5	NA	$bla_{CTX-M-14}$ (40.8%), $bla_{CTX-M-15}$ (15.3%), $bla_{CTX-M-3}$ (3.1%), $bla_{CTX-M-55}$ (23%), $bla_{CTX-M-65}$ (2.6%)
Lee CH, et al. 2015, Taiwan [7]	50	0.5	128	0.06 - >256	$bla_{CTX-M-14}$ (50%), $bla_{CTX-M-15}$ (88.8%)
Matsumura Y, et al. 2016, Japan [8]	269	≤1	≤1	NA	$bla_{CTX-M-14}$ (42.4%), $bla_{CTX-M-15}$ (26.8%), $bla_{CTX-M-27}$ (30.9%)
Jung Y, et al. 2019, Korea [10]	93	1	4	NA	$bla_{CTX-M-1}$ (51.6%), $bla_{CTX-M-9}$ (40.9%)
Ngoi ST, et al. 2021, Malaysia [11]	50	0.5	32	0.125 - 64	bla_{CTX-M} and bla_{TEM} (34%), bla_{CTX-M} (24%), bla_{TEM} (20%)
Sakaeda K, et al. 2023, Japan [12]	158	0.125	0.5	NA	bla_{CTX-M} (97.5%), bla_{TEM} (32.9%), bla_{SHV} (1.3%)
Yap PSX, et al. 2023, Malaysia [13]	127	4	32	0.125 - >64	$bla_{CTX-M-1}$ (22%), $bla_{CTX-M-1}$ and bla_{TEM} (21.3%), bla_{TEM} (18.1%)
Yamashiro H, et al. 2023, Japan [14]	180	0.12	0.5	≤0.06 - >64	$bla_{CTX-M-9}$ (NA), $bla_{CTX-M-1}$ (NA), $bla_{CTX-M-2}$ (NA), $bla_{CTX-M-8}$ (NA)
Iio K, et al. 2024, Japan [15]	2615	≤1	≤1	≤1 - >32	NA

N, the number of strains; MIC_{50}, minimum concentration that inhibited the growth of 50% of the clinical isolates (μg/mL); MIC_{90}, minimum concentration that inhibited the growth of 90% of the clinical isolates (μg/mL); NA, Not applicable.

(B) Susceptibility of ESBL producing *Klebsiella pneumoniae*

Literature	N	MIC_{50}	MIC_{90}	MIC range	ESBL genotype
Yang Q, et al. 2015, China [2]	25	0.125	0.5	NA	$bla_{CTX-M-14}$ (36%), $bla_{CTX-M-15}$ (28%), $bla_{CTX-M-3}$ (16%) $bla_{CTX-M-55}$ (8%), $bla_{CTX-M-65}$ (8%), bla_{SHV} (36%)
Yang Q, et al. 2015, China [6]	124	0.064	0.25	NA	$bla_{CTX-M-14}$ (34.7%), $bla_{CTX-M-15}$ (16.1%), bla_{SHV} (8.9%), $bla_{CTX-M-3}$ (7.3%)
Lee CH, et al. 2015, Taiwan [7]	30	0.25	256	0.06 - >256	$bla_{CTX-M-14}$ (31.3%), bla_{SHV-12} (75%)
Jung Y, et al. 2019, Korea [10]	83	0.5	16	NA	$bla_{CTX-M-1}$ (75.9%), $bla_{CTX-M-9}$ (20.5%)
Ngoi ST, et al. 2021, Malaysia [11]	50	1	8	0.06 - 64	bla_{CTX-M}, bla_{TEM} and bla_{SHV} (68%), bla_{CTX-M} and bla_{SHV} (12%)
Yamashiro H, et al. 2023, Japan [14]	33	0.12	0.25	≤0.06 - 4	$bla_{CTX-M-9}$ (NA), $bla_{CTX-M-1}$ (NA), $bla_{CTX-M-2}$ (NA), $bla_{CTX-M-8}$ (NA)
Iio K, et al. 2024, Japan [15]	1258	≤1	≤1	≤1 - >16	NA

(C) Susceptibility of ESBL producing *Escherichia coli and Klebsiella pneumoniae*

Literature	N	MIC_{50}	MIC_{90}	MIC range	ESBL genotype
Wi YM, et al. 2025, Korea [16]	29	0.5	16	0.25 - 64	$bla_{CTX-M-1}$ (89.7%), $bla_{CTX-M-9}$ (24.1), bla_{SHV} (48.3%), bla_{TEM} (41.4%)

N, the number of strains; MIC_{50}, minimum concentration that inhibited the growth of 50% of the clinical isolates (µg/mL); MIC_{90}, minimum concentration that inhibited the growth of 90% of the clinical isolates (µg/mL); NA, Not applicable.

(D) Susceptibility of ESBL producing *Escherichia coli, Klebsiella pneumoniae and Proteus species*

Literature	N	MIC_{50}	MIC_{90}	MIC range	ESBL genotype
Partina I, et al. 2016, Russia [9]	164	1	64	0.125 - >64	NA

(E) Susceptibility of ESBL producing *Proteus mirabilis*

Literature	N	MIC_{50}	MIC_{90}	MIC range	ESBL genotype
Yang Q, et al. 2015, China [2]	46	0.125	0.5	NA	$bla_{CTX-M-14}$ (37%), $bla_{CTX-M-15}$ (21.7%), $bla_{CTX-M-3}$ (4.3%) $bla_{CTX-M-55}$ (6.5%), $bla_{CTX-M-65}$ (52.2%), bla_{SHV} (2.2%)
Yang Q, et al. 2015, China [6]	81	0.125	0.5	NA	$bla_{CTX-M-65}$ (40.7%), $bla_{CTX-M-14}$ (29.6%)

N, the number of strains; MIC_{50}, minimum concentration that inhibited the growth of 50% of the clinical isolates (µg/mL); MIC_{90}, minimum concentration that inhibited the growth of 90% of the clinical isolates (µg/mL); NA, Not applicable.
